# Protocol for a randomized controlled trial with a stepped care approach, utilizing PrEP navigation with and without contingency management, for transgender women and sexual minority men with a substance use disorder: Assistance Services Knowledge-PrEP (A.S.K.-PrEP)

**DOI:** 10.1186/s13722-024-00482-6

**Published:** 2024-11-09

**Authors:** Cathy J. Reback, Raphael J. Landovitz, David Benkeser, Ali Jalali, Steven Shoptaw, Michael J. Li, Raymond P. Mata, Danielle Ryan, Philip J. Jeng, Sean M. Murphy

**Affiliations:** 1 Friends Research Institute, Inc, Los Angeles, CA USA; 2 Center for HIV Identification, Prevention and Treatment Services, Department of Family Medicine, University of California, Los Angeles, Los Angeles, CA USA; 3 Center for Behavioral and Addiction Medicine, Department of Family Medicine, University of California, Los Angeles, Los Angeles, CA USA; 4 Division of Infectious Diseases, University of California, Los Angeles, Los Angeles, CA USA; 5 Department of Biostatistics and Bioinformatics, Rollins School of Public Health, Emory University, Atlanta, GA USA; 6 Department of Population Health Sciences, Weill Cornell Medicine, New York, NY USA; 7 Center for Health Economics of Treatment Interventions for Substance Use Disorder, HCV, and HIV (CHERISH), New York, NY USA

**Keywords:** HIV, Substance Use Disorder, PrEP care Continuum, Transgender women, Sexual minority men

## Abstract

**Background:**

In the United States, most (~ 70%) annual newly diagnosed HIV infections are among substance-using sexual minority men (SMM) and gender minority transgender women (trans women). Trans women and SMM are more likely to report or be diagnosed with a substance use disorder (SUD) than their cisgender or heterosexual counterparts and the presence of an SUD substantially increases the risk of HIV infection in both groups. Although Pre-Exposure Prophylaxis (PrEP) is highly effective, initiation, adherence, and persistence are exclusively behavioral outcomes; thus, the biomedical benefits of PrEP are abrogated by substance use. SUD is also associated with reduced quality-of-life, and increased overdose deaths, utilization of high-cost healthcare services, engagement in a street economy, and cycles of incarceration.

**Objective:**

To determine the optimal (considering efficacy and cost-effectiveness) strategy for advancement along the PrEP Care Continuum among trans women and SMM with an SUD.

**Methods:**

This study will implement a randomized controlled trial, evaluating two Stepped Care approaches involving *A.S.K.-PrEP* vs. standard of care (SOC) to determine optimal intervention strategies for trans women and SMM with an SUD (*N* = 250; *n* = 83 trans women; *n* = 167 SMM) for advancement along the PrEP Care Continuum. Participants will be randomized (3:1) to Stepped Care (*n* = 187) or SOC (*n* = 63). Participants in the Stepped Care arm will be assessed at 3-months for intervention response; responders will be maintained in *A.S.K.-PrEP*, while non-responders will receive added attention to their SUD via Contingency Management (CM). Non-responders will be re-randomized (1:1) to either (a) receive *A.S.K.-PrEP* + CM, or (b) shift the primary focus to their SUD (CM alone).

**Results:**

Recruitment and enrollment began in May 2023. Recruitment will span approximately 36 months. Data collection, including all follow-up assessments, is expected to be completed in April 2027.

**Discussion:**

Trans women and SMM with an SUD have the two highest HIV prevalence rates in the United States, which underscores the urgent need for effective measures to develop scalable behavioral interventions that can encourage advancement along the PrEP Care Continuum. To improve public health, researchers must identify scalable and cost-effective behavioral interventions to promote PrEP initiation, adherence, and persistence among trans women and SMM who use substances.

**Trial registration:**

This trial has been registered at ClinicalTrials.gov under the number NCT05934877.

## Background

Tenofovir disoproxil fumarate/emtricitabine (TDF/FTC), tenofovir alafenamide/emtricitabine (TAF/FTC), and Cabotegravir to prevent HIV are highly effective when taken as prescribed [1]. In spite of this scientific advance, and scale-up of treatment efforts for persons with HIV (PWH) to reduce sexual transmissibility, the US still has ~ 35,000 annual HIV diagnoses, the majority of which occur among substance-using transgender women (hereafter: trans women) and sexual minority men (SMM) [2], groups that also evidence poor PrEP adherence. In the United States, trans women are the group at greatest risk for HIV, with an estimated HIV prevalence of 22–28% [3], while SMM have an estimated HIV prevalence of 15% [4]. These alarmingly high HIV prevalence rates constitute a call for action to determine scalable behavioral interventions to promote PrEP initiation, adherence, and persistence among trans women and SMM with a substance use disorder (SUD). A majority of trans women who initiate PrEP do not achieve prevention-effective adherence [5], that is sufficient biomarker-measured adherence required to achieve maximal levels of clinical HIV prevention, and similar outcomes are observed among high-risk SMM [6, 7]. Elevated seroconversion rates among trans women and SMM persist because, although PrEP is highly effective, PrEP initiation, adherence, and persistence – whether taking a pill or getting an injection – are exclusively behavioral outcomes that, by definition, mediate all biomedical benefits of PrEP; these benefits can be diminished in the setting of active use of stimulants and other substances [5, 7].

### Substance use impedes PrEP engagement among trans women and SMM

Numerous studies demonstrate that SUD is a major disruptor of medication adherence; whereas, reduced substance use is associated with increased medication adherence, and mitigation of HIV drug and sexual risk behaviors [8–10]. Among trans women and SMM, stimulant use in particular has been shown to interfere with PrEP initiation, adherence, and persistence [11–13]. Such findings are particularly concerning because trans women and SMM are not only more likely to report or be diagnosed with a current SUD than their cisgender or heterosexual peers [14, 15], but the presence of an SUD also substantially increases risk of HIV infection in both groups [16–18].

Trans women may face structural and interpersonal transmisogyny in the forms of mistreatment or hindered to access to opportunities and services [19, 20]. This can increase their risk of SUD and related health disparities, including housing instability and poverty [21, 22], engagement in sex work [23, 24], cycles of incarceration [25, 26], intimate partner violence [27, 28], sexual violence [29, 30], and mental health disorder(s) [30]. Low health literacy, sex work, and the experience of stigma or lack of gender-related social support are further known to increase medical mistrust and decrease HIV-related medication adherence and care engagement, especially in the presence of comorbid SUD [31, 32]. Similarly, SMM may experience structural and interpersonal forms of homophobia [33, 34], which may underlie SUD and comorbid housing instability [33], sexual violence [35, 36], physical violence [37, 38], mental health disorder(s) [15], and suicidality [39]. SUDs, mental health disorders, low health literacy, poverty, and traumatic stress have also all been shown to negatively affect HIV-related medication adherence among SMM [40, 41]. Due to these health disparities, evidence indicates that trans women and SMM exhibit poor knowledge and slow uptake of PrEP [42, 43], and when PrEP initiation does occur, suboptimal medication adherence is common [5, 7].

In general, SUD is associated with reduced quality-of-life, suboptimal utilization of primary and ambulatory care services, and increased overdose deaths, utilization of high-cost healthcare services (e.g., emergency department and inpatient encounters), engagement in the street economy, and criminal-legal activity [44–47]. The discounted average lifetime healthcare cost per United States PWH is estimated to be $368,000 (2020 USD), and healthcare costs have been shown to be 1.2–1.6 times higher for those with comorbid SUD [48]. The discounted average lifetime cost of non-healthcare SUD-related consequences (e.g., premature mortality, criminal-legal) could be an additional ~$2 million [47]. Thus, strategies that improve PrEP initiation, adherence, and persistence, as well as treatment for SUD, are critically important for personal, public, and national-economic health.

This study is a randomized clinical trial to evaluate the efficacy and cost-effectiveness of the Assistance Services Knowledge-PrEP (A.S.K.-PrEP) intervention, a PrEP navigation intervention with Short Message Service (SMS) support [49], embedded in a Stepped Care design that incorporates Contingency Management (CM) to reward improved SUD outcomes among non-responders. The overarching objective of the study is to determine the optimal (considering efficacy and cost-effectiveness) strategy for advancement along the PrEP Care Continuum among trans women and SMM.

## Methods

### Research aims

**Fig. 1:**
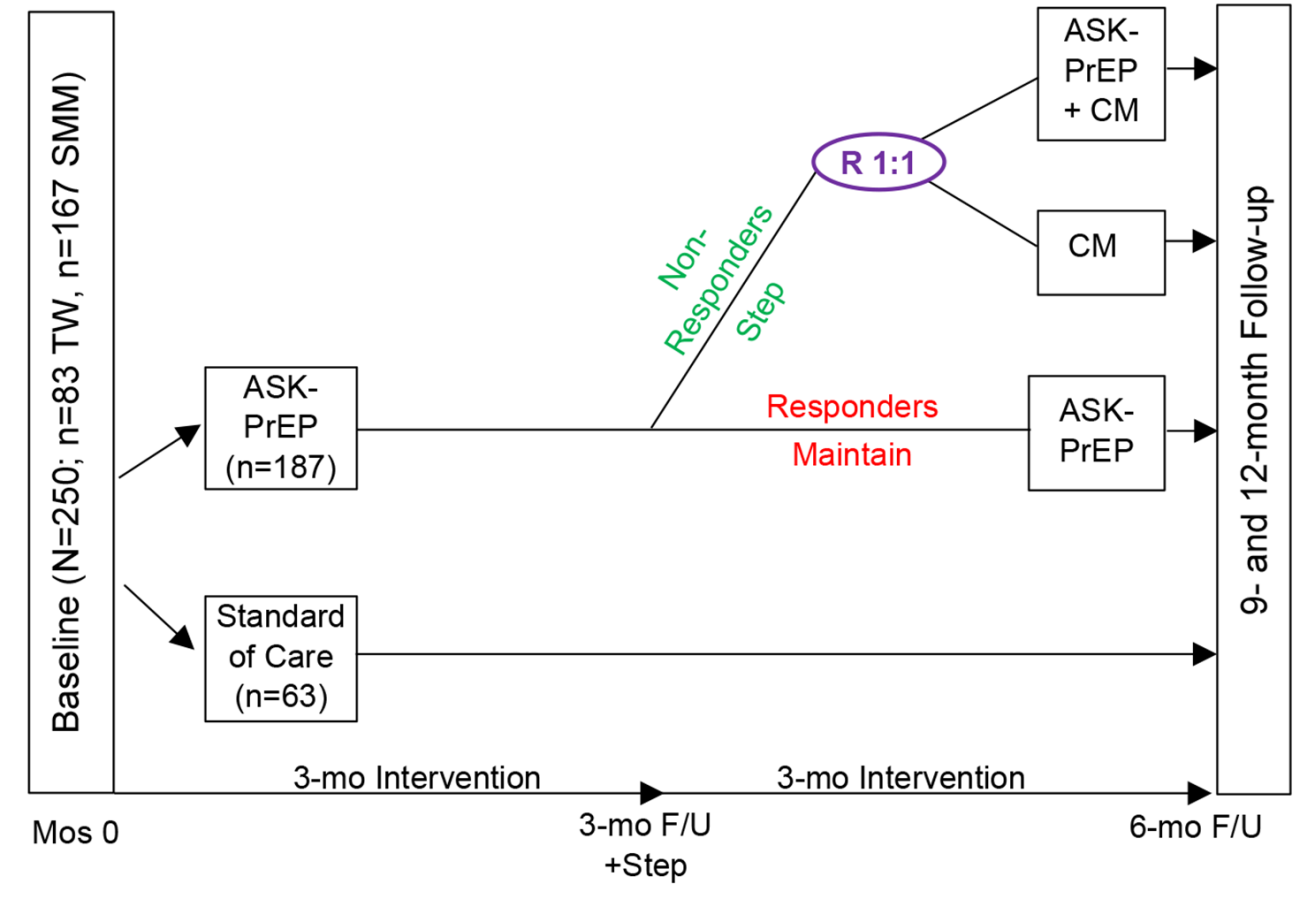
Schematic of ASK-PrEP Stepped Care, Randomized Controlled Trial Design

The specific objectives of the *A.S.K.-PrEP* study are to evaluate (1) a Stepped Care approach for promoting advancement along the PrEP Care Continuum (based on PrEP initiation, adherence, and persistence), and reductions in substance use, and (2) the cost-effectiveness of the *A.S.K.-PrEP* intervention in a Stepped Care approach with added attention to SUD, via CM, among trans women and SMM with an SUD (see Fig. 1).

The trial will recruit 250 HIV-negative trans women and SMM with an SUD (*n* = 83 trans women; *n* = 167 SMM); participants will be randomized (3:1) to *A.S.K.-PrEP* (*n* = 187), or Standard of Care (SOC; PrEP education, information, and referrals) (*n* = 63). Participants in the *A.S.K.-PrEP* Stepped Care arm will receive 5 PrEP navigation sessions within 3 months and will be assessed at 3-months for intervention response; responders will maintain the *A.S.K.-PrEP* intervention, while non-responders will be re-randomized (1:1) to either add attention to their SUD (*A.S.K.-PrEP* + CM), or shift attention to their SUD (CM alone). The study uses repeated assessments at baseline and at 3-, 6-, 9-, and 12-months post-enrollment; all assessments will be administered to participants regardless of their participation or retention. All intervention content is tailored to trans women and SMM with an SUD. Participants may choose to initiate daily oral PrEP (TDF/FTC or TAF/FTC) or long-acting injectable Cabotegravir [10].

The specific aims of the research include the following:

#### Aim 1:

Evaluate the efficacy of a Stepped Care approach promoting advancement along the PrEP Care Continuum (initiation, adherence, persistence), and reductions in substance use among trans women and SMM with an SUD, relative to an SOC condition.

#### Hypothesis

*a*: Participants randomized to the *A.S.K.-PrEP* Stepped Care arm will achieve protective PrEP adherence, persistence, and reductions in substance use at greater rates over time, relative to participants in the SOC arm.

#### Hypothesis

*b*: Among participants randomized to the *A.S.K.-PrEP* Stepped Care arm, participants in arms containing CM will produce superior outcomes in PrEP adherence and persistence and greater reductions in substance use compared to participants in arms not containing CM.

#### Aim 2:

Estimate the cost of implementing and sustaining each intervention (Stepped Care with: a] *A.S.K.-PrEP* + CM; b] CM alone) and incorporate these costs into a comprehensive cost-effectiveness analysis to determine the value of each intervention relative to SOC, and to each other, from the healthcare-sector, state-policymaker, and societal perspectives.

#### Hypothesis 2

The Stepped Care approach with *A.S.K.-PrEP* + CM will be the most cost-effective intervention, despite its relatively high sustainment cost, due to its greater effectiveness resulting in larger improvements along the PrEP Care Continuum, and in SUD, thereby generating: (a) reduced utilization of high-cost healthcare, safety-net, and criminal-legal resources; and (b) increased productivity, time free from primary substance, and quality-adjusted life-years (QALYs). Stepped Care with CM alone will be the next best “value” for the same reasons.

#### Secondary Aim 1:

Estimate the individual effects of specific substances (e.g., methamphetamine vs. opioids), routes of administration (injection vs. non-injection), severity of SUD (mild, moderate, severe), social and structural determinants of health (e.g., poverty, housing insecurity, food scarcity, educational attainment, lack of insurance), and differing individual-level characteristics (e.g., sexual/gender identity, racial/ethnic identity, age) as moderators of outcomes among trans women and SMM with an SUD.

#### Exploratory Aim 1:

Evaluate intervention engagement (number of *A.S.K.-PrEP* navigation sessions in the initial 3-months) and intervention response (responders vs. non-responders) by chosen PrEP modality (i.e., oral daily [TDF/FTC or TAF/FTC] or long-acting injectable cabotegravir).

### Randomization ratio and power calculation

The 3:1 initial randomization ratio was selected as a means of increasing the likelihood that an adequate number of participants receive each of the two Stepped Care approaches. Because our second-stage randomization only occurs in non-responders, there is some uncertainty at baseline as to how many participants will ultimately be in each of the three arms. We used Monte Carlo simulations to establish under a range of scenarios that a sample size of *n* = 250 and the selected randomization ratios would ensure at least 80% power to detect a clinically meaningful difference of 20% or more in the proportion persistence/adherence under intervention vs. SOC.

### Community Advisory Board (CAB)

Two long-standing and ongoing CABs, one trans-specific and one SMM-specific, participate in all aspects of the study. The CAB members are requested to provide feedback on all stages of study development, implementation, recruitment strategies, interpretation of findings, and address any challenges encountered. Both CABs are multi-cultural and composed of individuals both living with and at risk for HIV, social service providers, community members, consumers, evaluation professionals, and influencers.

### Interventions

#### A.S.K.-PrEP intervention

*A.S.K.-PrEP* is a PrEP navigation intervention with SMS support. The navigation component is based on mechanisms of the Reasoned Action Approach [50], and the SMS support component is based on Social Support Theory [51]. The *A.S.K.-PrEP* intervention was adapted from Anti-Retroviral Treatment and Access to Services (ARTAS), the 5-session Centers for Disease Control and Prevention (CDC) evidence-based intervention, delivered over 3 months, for linking PWH into HIV care. Formative work was conducted to modify ARTAS to focus on linking high-risk, HIV-negative trans women and SMM into PrEP care [49]. In Session 1, the PrEP navigator uses the Needs and Barriers Assessment (NBA) to identify those pertaining to PrEP care, including substance use and behavioral health needs; adherence goal(s); and methods to achieve adherence. PrEP (TDF/FTC, TAF/FTC, Cabotegravir) and clinic options are discussed. In Sessions 2–5, PrEP navigators use the information from the NBA to help participants overcome barriers to PrEP adherence, and work with participants to address their SUD and need for additional auxiliary services.

**Fig. 2:**
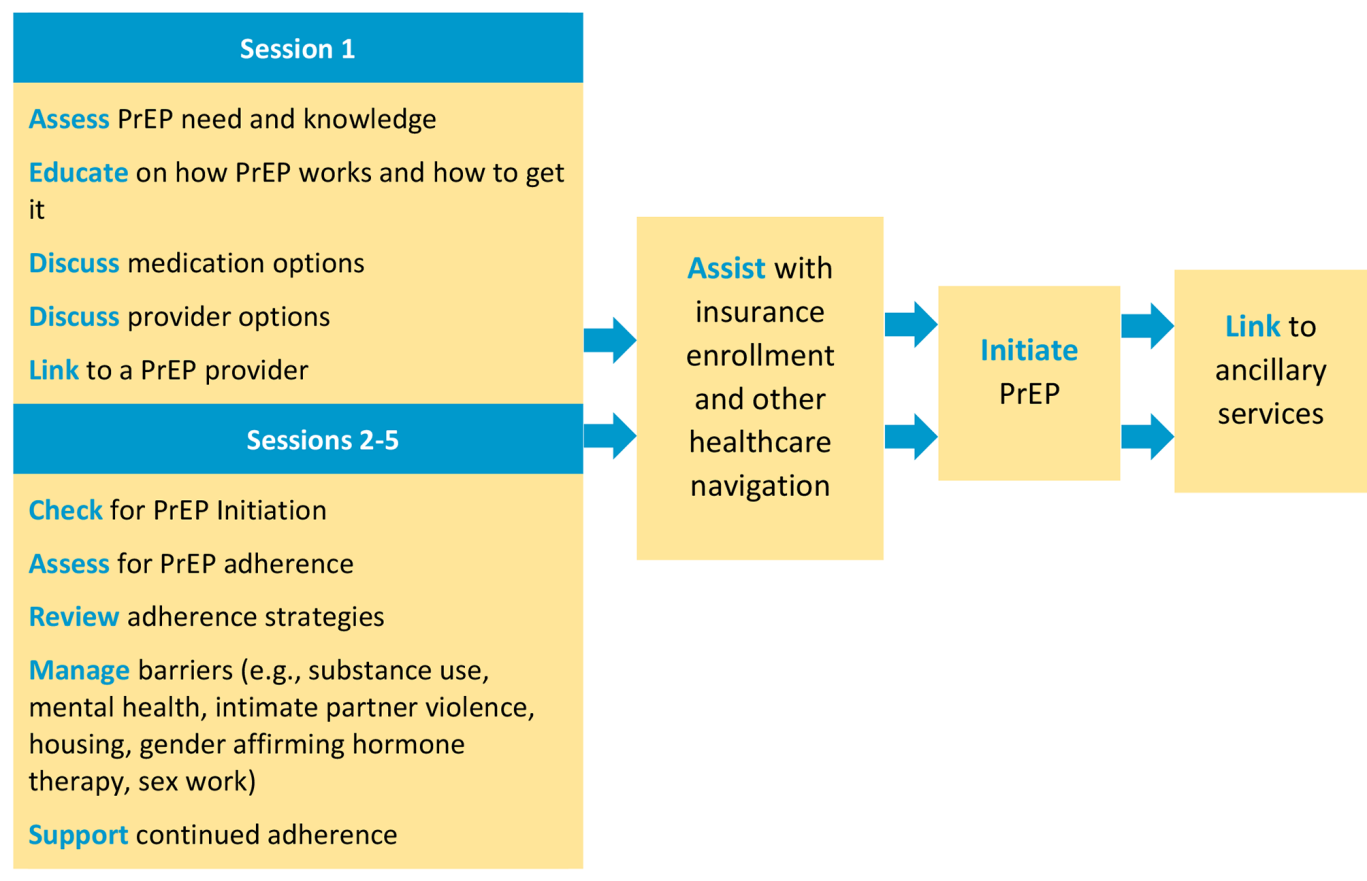
PrEP Navigation Delivery System

A shorter version of the NBA (NBA-Lite) is administered at the beginning of Sessions 2–5 as a check-in and to assess progress through the PrEP Care Continuum; participant-centered treatment plans are reviewed and revised as needed. The participant-centered dialogical strategies are premised on Reasoned Action Approach: (1) identify barriers to PrEP, including substance use; (2) identify the participant’s readiness to address their SUD and link into other auxiliary needed services; and (3) increase the participants’ skills and self-efficacy in working with PrEP providers and other social service and treatment facilities. The PrEP navigator will link participants into SUD treatment and other ancillary behavioral health and support services, according to their unique barriers, with the ultimate goal of PrEP initiation, adherence, and persistence; services may include mental healthcare, counseling for intimate partner violence, food insecurity, housing instability, hormone therapy, among others. Discussions of PrEP adherence, reductions in substance use, substance use treatment, and other behavioral health concerns are discussed throughout the 5 sessions (see Fig. 2). All PrEP navigators are all peers, with past lived experiences directly related to the participants.

#### SMS support

One PrEP social support text message (see Table 1) is transmitted weekly; participants may choose to have the text messages delivered via cell phone or email. The automated text message delivery system is developed in and delivered by TextMagic on a pre-programmed schedule. To maintain interest and enthusiasm for the intervention, participants receive 12 trans- or 12 SMM-specific messages during the 3-month intervention period. Participants that continue with the A.S.K.-PrEP intervention for an additional 3 months receive the same SMS support intervention. Participants are asked to notify a research assistant immediately if they lose their cell phone or changed their phone number.

**Table 1:** Sample text messages for PrEP adherence geared towards trans women and SMM

Trans Women-specific Text Messages	SMM-specific Text Messages
“Hormones are safe to take with PrEP! PrEP is safe to take with hormones! You can do both!”	“If you can see your friends, you can get your PrEP”
“Hey gurl, you can prioritize PrEP, even if you’re high.”	“You can prioritize PrEP, even if you’re high”
“You can take care of yourself and your trans community by taking PrEP.”	“Take care of yourself, take care of your sex partners, take PrEP.”

#### Contingency management (CM)

CM is a behavioral economics intervention that posits the application of contingencies to motivate individuals toward health-promoting behavior change [52, 53]. Behavioral economics incorporates direct and immediate reinforcement – the primary construct in the operant form of learning theory – whereby behaviors are learned through rewards. CM uses an operant reinforcement schedule that provides increasingly valuable rewards for consecutive urine samples that are nonreactive to the participant’s targeted SUD. As successive nonreactive urine samples are achieved and voucher points increase, so does one’s sense of self-efficacy to continue providing nonreactive urine samples.

At the 3-month follow-up assessment, non-responders (see Table 2) are stepped and re-randomized to CM, either in concert with *A.S.K.-PrEP* or alone. At the first CM session, a research assistant provides a 15-minute orientation to the CM procedures, which includes an explanation of the progressive, voucher-based reinforcement schedule. Thereafter, participants will meet with a research assistant thrice weekly to provide a urine sample. The CM protocol can be simplified into these guidelines: (1) participants receive a $2.50 voucher for the first nonreactive urine sample for their targeted SUD; (2) participants receive a voucher reflecting an incremental increase of $0.50 for each subsequent nonreactive urine sample; (3) participants receive a bonus of $7.50 for each 3 consecutive nonreactive urine samples; (4) vouchers are redeemable at any time for goods or services, selected by the participant, that support a healthy and pro-social lifestyle, such as groceries, camera equipment, clothing, bicycle; (5) participants who produce a urine sample that is reactive to their targeted SUD, or who fail to submit a urine sample, do not receive a voucher for that particular CM visit and their subsequent voucher value is reduced to the initial $2.50; and (6) a rapid reset procedure allows participants to return to their place in the escalating CM schedule after producing 3 consecutive nonreactive urine samples. Participants who complete the CM intervention without a reactive urine sample earn $495 in vouchers (see Table 3). If a participant is diagnosed with a SUD for two or more substances, they are asked, “Which drug do you use most despite negative consequences?” and that drug is targeted in the CM intervention.

**Table 2:** Step criteria at 3-month follow-up

**Step Criteria Based on PrEP:**
Has not initiated PrEP (either Truvada, Descovy or Cabotegravir)
On oral-daily PrEP but self-reports non-adherence (missed 4 or more days in a row) in past 3 months
On long-acting injectable but has not received 2nd dose within +/- 7 days of 28 days (i.e., 35 days) after 1st dose (only check this if 3-month follow-up takes place 35 days or more after 1st dose)
**Step Criteria Based on Substance Use**:
Self-reported drug use of the targeted SUD diagnosed at baseline for 1 (or more) day(s) in the past 14 days
Positive urine drug screen for targeted SUD diagnosed at baseline

**Table 3:** Contingency management (CM) Voucher-based escalating reinforcement schedule

Week #	Mon	Wed	Fri	Bonus	Weekly Totals	Total Earned
**Week 1**	$2.50	$3.00	$3.50	$7.50	$16.50	$16.50
**Week 2**	$4.00	$4.50	$5.00	$7.50	$21.00	$37.50
**Week 3**	$5.50	$6.00	$6.50	$7.50	$25.50	$63.00
**Week 4**	$7.00	$7.50	$8.00	$7.50	$30.00	$93.00
**Week 5**	$8.50	$9.00	$9.50	$7.50	$34.50	$127.50
**Week 6**	$10.00	$10.50	$11.00	$7.50	$39.00	$166.50
**Week 7**	$11.50	$12.00	$12.50	$7.50	$43.50	$210.00
**Week 8**	$13.00	$13.50	$14.00	$7.50	$48.00	$258.00
**Week 9**	$14.50	$15.00	$15.50	$7.50	$52.50	$310.50
**Week 10**	$16.00	$16.50	$17.00	$7.50	$57.00	$367.50
**Week 11**	$17.50	$18.00	$18.50	$7.50	$61.50	$429.00
**Week 12**	$19.00	$19.50	$20.00	$7.50	$66.00	$495.00
**Maximum CM Payout**:						$495.00

#### A.S.K.-PrEP + contingency management

Participants stepped and randomized into *A.S.K.-PrEP* + CM receive the same *A.S.K.-PrEP* and CM interventions (described above), but in concert to increase intensity and simultaneously address SUD, and PrEP initiation, adherence, and persistence.

#### Standard of care arm

The SOC arm provides PrEP education, information, and referrals. Those randomized to the SOC arm at baseline receive a 20- to 30-minute educational session on PrEP; the CDC “PrEP 101” and “PrEP Medication Guide” pamphlets [54, 55]; and a list of clinics that provide PrEP in LAC. For those randomized into the SOC arm, the same educational session is repeated following the 3-month follow-up assessment visit. A research assistant provides the PrEP education, information and referrals at baseline and the 3-month follow-up assessment visit.

### Sample

The inclusion criteria for participation are: (1) self-identified trans woman or SMM (including trans man/trans masculine male who have sex with men); (2) age 18 years or older; (3) verified HIV negative; (4) identified as “high risk” for HIV based on the Los Angeles County (LAC) criteria of: (a) sex without a condom, (b) methamphetamine use, (c) sex with an HIV-positive partner, or (d) injection drug use [56]; 5) SUD (injection and non-injection) verified by the Diagnostic and Statistical Manual of Mental Disorders, Fifth Edition (DSM-5) (excluding cannabis use disorder alone, as current research has demonstrated that cannabis use only is not an HIV risk) [57]; 6) willing to provide informed consent; and 7) willing to comply with study requirements. Additional inclusion criteria for those who previously initiated PrEP are: missed 4 or more doses of oral-daily PrEP during any week in the previous 30 days, and/or no PrEP care visits in the past 3 months. Individuals that do not meet all criteria or are unable to pass an informed consent quiz are excluded. Should a participant be identified as having kidney or liver dysfunction sufficient to contraindicate PrEP use (creatinine clearance < 60 mL/min), that individual is withdrawn from study participation, but may re-screen for eligibility once adequate renal and/or liver function has been established and medically documented (≥ 60 mL/min).

### Procedures

#### Recruitment

Six recruitment strategies are being utilized to ensure enrollment targets are met and a diversity of participants are enrolled: (1) *online*: banners advertisements and digital flyers are placed through geo-mapping on relevant websites and social media platforms; (2) *print media*: advertisements are placed in local print media for trans women and SMM; (3) *outreach*: two research assistants conduct outreach in identified areas where trans women and SMM who use substances congregate, including bars/clubs, motels, parks, street corners, mini markets, boutiques, wig shops, electrolysis offices, salons, lingerie stores, bathhouses, and sex clubs (outreach locations are continually modified through ongoing community mapping and input from the [CAB]); (4) *posters and club cards*: are displayed/distributed throughout the study site and collaborating clinics, organizations in the LAC area serving trans women or SMM, and at dance clubs, bars, and community events; (5) *in-reach*: several service programs operate at the study site that cater to trans women and SMM who use substances; and (6) *long-chain referral*: current study participants are asked to recruit a maximum of 3 potential new participants, and then receive an incentive when an eligible potential participant enrolls.

#### Randomization

Stratified block randomization with random block sizes is used to assign participants to each of the two initial study arms (stepped care vs. SOC, 3:1 ratio) and to assign non-responding participants at the 3-month visit to the two stepped care modalities (*A.S.K.-PrEP* + CM vs. CM alone, 1:1 ratio). To ensure balance with respect to certain covariates, participants are grouped into four strata: (1) SMM < 35 years and/or African American/Black; (2) SMM > 34 and non-African American/Black; (3) trans women < 35 years and/or African American/Black; (4) trans women > 34 and non-African American/Black. Blocked randomization lists are generated separately for each strata by generating a block of random size (block size = 4, 8, or 12 at baseline; block size = 2, 4, or 6 at 3 months).

#### Incentives

All participants are compensated $15 for the screener; $50 for the baseline and 3-month follow-up assessment, with an additional $25 bonus for completing within +/- 7 days of the exact 3-month due date; $75 for the 6- and 9-month follow-up assessment; and $100 for 12-month follow-up assessment. Incentives are provided in gift cards; participants are not given cash. Participants are offered a drink (e.g., water, soda, or juice) and a snack (e.g., granola bar, box of raisins, bag of chips) during each assessment data visit.

### Measures

All assessments have been used in prior studies; some have been modified and tailored by our group for trans women and SMM who use substances (see Table 4) [23, 58]. Assessments were chosen to minimize participant burden while addressing study aims, and are estimated to take 45–60 min at baseline, and 30–45 min at each follow-up time point. All assessments will be collected using Audio Computer-Assisted Self-Interview.

**Table 4:** Assessments, corresponding aims, variables, and Time points

Assessment Instrument	Aims	Variables	Screening	Baseline	3-mo F-U	6-mo F-U	9-mo F-U	12-mo F-U
MINI DSM-5	1,S	substance use disorder	x					
HIV Rapid Antibody Test	1,S	HIV status		x				
*ASK-PrEP* Admission/Follow-up Assessment	1,S	demographics; individual-level characteristics; social/structural determinants		x	x	x	x	x
Timeline Follow-Back*****	1,S	Current substance use		x	x	x	x	x
NBA* / NBA-Lite**	1,S, E	needs, barriers and facilitators						
PrEP Care Continuum	1,S, E	PrEP initiation, adherence, persistence; PrEP advancement		x	x	x	x	x
HIV-ASES	1	components of PrEP self-efficacy		x	x	x	x	x
DBS***	1,S, E	oral daily PrEP adherence and persistence		x	x	x	x	x
Urine Drug Screen	1,S	current substance use		x	x	x	x	x
HIV Ag/Ab and Viral Load Test	1,S, E	HIV seroconversion			x	x	x	x
Electronic Health Records	1,S, E	PrEP care, PrEP prescriptions, PrEP advancement, liver and kidney functions		x	x	x	x	x
DATCAP****	2	intervention cost		x	x	x	x	x
NMOS	2	resource costs		x	x	x	x	x
PROPr	2	HRQoL, QALYs		x	x	x	x	x
Locator Form	n/a			x	x	x	x	

*Administered during first PrEP navigation session only.

**Administered during all subsequent PrEP navigation sessions.

***With participants that report PrEP use only.

****Administered to study staff only.

*****Administered weekly, on Wednesdays, during the Contingency Management intervention.

#### Diagnostic and statistical manual-5 (DSM-5)

The DSM-5 assesses symptom criteria to ensure potential participants meet eligibility for a current SUD (mild, moderate, or severe). Findings are used to describe the sample, control for SUD severity, and examine the effect of SUD(s) severity as it pertains to advancement along the PrEP Care Continuum.

#### Rapid HIV antibody test

Potential participants are administered a rapid HIV antibody test (> 95% sensitivity, > 99% specificity) [59] during the screening process to verify HIV negative status. If the test is reactive, the potential participant is referred for additional evaluation and treatment.

#### ASK-PrEP admission/follow-up assessment

The full assessment is administered at baseline, and an abbreviated version is administered at all follow-up time points. The assessment collects demographics, substance use history, housing status, food security, educational attainment, HIV sexual risks, family and social history, legal status, incarceration history, sexually transmitted infections, hepatitis history, intimate partner violence, and gender confirmation procedures (for trans women participants only).

#### Timeline follow-back (TLFB)

TLFB [60] measures self-reported frequencies, quantities, and routes of administration of substances used. The TLFB is administered at baseline and at all follow-up time points with a 14-day recall period, and weekly with a 7-day recall period, alongside urine drug screens for those stepped to CM.

#### Needs and barriers assessment (NBA)

The NBA identifies behavioral health cofactors that may impede one’s ability to initiate and adhere to PrEP care (e.g., housing instability, poverty, transportation, legal restrictions, etc.). The NBA is administered during the *A.S.K.-PrEP* sessions to assist PrEP navigators with developing individualized participant-centered treatment plans, including SUD treatment options, to assess progress through the PrEP Care Continuum, and to reset priorities and identify possible new barriers. The shorter NBA-Lite is utilized in Sessions 2–5 to reassess needs and barriers as they change during intervention participation.

#### PrEP care continuum

In addition to biomarkers, participants self-report PrEP readiness: initiation, missed doses in the previous 4 days and 30 days (oral daily only), self-perceived ability to adhere, and structural-/individual-level barriers to adherence and persistence. The instrument includes three sections: (1) currently on PrEP, (2) formerly on PrEP, and (3) never on PrEP.

#### HIV Treatment Adherence Self-Efficacy Scale (HIV-ASES)

The HIV-ASES assesses participants’ self-efficacy to adhere to their HIV medication regimen (Cronbach’s alpha is routinely > 0.90) [61]. The HIV-ASES is lightly adapted to participants’ level of confidence that they can maintain PrEP adherence.

#### Dried blood spot (DBS)

DBS analysis for intra-erythrocytic TFV-DP (tenofovir-diphosphate; for participants using TDF or TAF-based PrEP) is performed by Dr. Peter Anderson’s laboratory at the University of Colorado, Denver to assess PrEP adherence. Results provide an assessment of average weekly adherence to oral TDF/FTC or TAF/FTC over the past 60–90 days (r2 ≥ 0.96 when regressed against plasma) [62]. No biomarker of Cabotegravir adherence is required, rather documentation of attendance at the clinic visit and administration of the injection at 2-month intervals is sufficient confirmation of adherence, given that the injection is directly observed.

#### Urine drug screen

Urine samples are collected via a 12-panel FDA-approved urine test cup by Confirm Biosciences, San Diego, CA to capture current use of various drug at varying detection times and concentration cut-offs: amphetamines (2–4 days, 1,000 ng/mL), barbiturates (4–7 days, 300 ng/mL), buprenorphine (103 days, 10 ng/ML), benzodiazepines (3–7 days, 300 ng/mL), cocaine (2–4 days, 300 ng/mL), methamphetamine (3–5 days, 1,000 ng/mL), MDMA (1–3 days, 500 ng/mL), opiates/morphine (2–4 days, 2,000 ng/mL), methadone (3–5 days, 1,000 ng/mL), oxycodone (2–4 days, 100 ng/mL), phencyclidine (7–14 days, 25 ng/mL), and cannabis (2–30 days, 50 ng/mL) [63].

#### HIV testing via laboratory-based hiv antigen/antibody test

At all follow-up visits, participants are tested by a laboratory-based (performed on phlebotomized blood) antigen/antibody test as the primary assessment of HIV serostatus and HIV-1 RNA (100% sensitivity, 99.5% specificity). If a participant provides laboratory results via Electronic Health Records from a PrEP care visit in the previous 3 months, the laboratory-based HIV antigen/antibody test is forgone.

#### Electronic health records (EHRs)

Research assistants work with participants to show them how to access their EHR through their provider’s portal. EHRs are utilized to record HIV status, retention in PrEP care, PrEP prescriptions, kidney and liver function to ensure that PrEP is well tolerated, and viral load.

#### Drug abuse treatment cost analysis program (DATCAP)

Resources required to implement and sustain each intervention are identified via microcosting analysis, which will entail the collection of administrative data, whenever feasible, along with semi-structured interviews with relevant staff members. The microcosting analysis is guided by a tailored version of the DATCAP, a standardized, customizable tool designed to capture intervention resources in a manner conducive to estimating the associated costs, across diverse settings [64].

#### Non-study medical and other services (NMOS)

Utilization of healthcare services are self-reported using time-anchoring methodology via the NMOS form. Healthcare services encompass non-study: inpatient, outpatient, and emergency department; SUD treatment medications; residential and outpatient SUD treatment days; hospital SUD detoxification days; and mental health treatment visits. Use of non-medical and other resources required for the economic evaluation from state-policymaker and societal perspectives (e.g., criminal-legal, labor productivity, travel time to medical care) is also self-reported and collected via the NMOS.

#### Patient-reported outcomes measurement information system (PROMIS)-preference (PROPr)

PROPr measures a participant’s health-related quality-of-life (HRQoL) across the following PROMIS domains: cognitive function abilities, depression, anxiety, fatigue, pain interference, pain intensity, physical function, sleep disturbance, and ability to participate in social roles and activities [65, 66]. PROPr is also capable of generating a health utility index value, from the participant’s scores for each domain, that represents the general United States population’s preference for the respondent’s current health state. PROPr has 5 levels for each domain, ranging from “no problems” to “extreme problems.” The health-utility value can range from − 0.022 to 1, where 0 represents death, 1 represents perfect health, and values below 0 represent states perceived to be worse than death.

### Statistical analysis/outcomes

*Specific Aim 1: Evaluate a Stepped Care approach for advancement along the PrEP Care Continuum (initiation, adherence, persistence) and reductions in substance use.*

The primary outcome for PrEP Care Continuum advancement is PrEP adherence and persistence. For those who initiate oral-daily PrEP, persistence is defined as a co-occurrence of ≥ 700 fmol/punch and ongoing use over a period of time (6 or 12 months) confirmed PrEP care medical visits. At each time point, those who initiate PrEP, attend a PrEP medical care visit in a given quarter, and evidence a DBS TFV-DP concentration of *≥* 700 fmol/punch are coded “PrEP adherent” and all others are coded as “non-adherent.” Participants who initiate PrEP, attend quarterly medical visits, and have a DBS TFV-DP concentration above the limit of quantification are coded “PrEP persistent”; all others are coded as “non-persistent”. For those who initiate long-acting injectable PrEP, adherence is defined as verified documentation of a Cabotegravir injection every 2 months, and persistence is defined as ongoing use over a period of time (6 or 12 months). Participants that discontinue PrEP due to elimination of their substance use and HIV sexual risk behaviors are categorized as persistent.

We quantify the primary intervention effects as the difference in proportion of participants who are adherent and persistent, comparing the Stepped Care intervention to SOC. We estimate this effect using longitudinal targeted minimum loss-based estimation (LTMLE) [67, 68]. This method appropriately accounts for the study design and the fact that only some participants are eligible for re-randomization, and accounts for predictive and prognostic time-varying participant-level covariates. Implementation of LTMLE involves estimating several sequential regressions modeling the probability of adherence and persistence as a function of time-varying covariates. In order to remove the potential for investigator bias in the analysis phase, we require that these regressions are fully defined a priori, which is challenging because model checking/re-fitting procedures cannot be employed while maintaining the study blind. Instead, we rely on the Super Learner for estimating these regressions [69]. The Super Learner is an ensemble machine learning approach that uses cross-validation to weight the contributions of several pre-specified candidate regression models. It can be used as a tool to pre-specify and fully automate the model selection process while remaining blinded to intervention assignments.

We study key secondary endpoints of PrEP initiation at 3-, 6-, 9-, and 12-months. PrEP initiation is operationalized dichotomously and is defined as confirmed acquisition of oral daily or long-acting injectable PrEP from a clinical provider. As with the primary analysis, we quantify treatment effects in terms of differences in probabilities of initiation and assess them via LTMLE and Super Learning.

Cumulative days of substance use are measured via TLFB, and urine drug screens, with the focus being the participant’s primary substance of concern. Self-reported substance-free days must be confirmed by non-reactive urine drug screen results; missing urine drug screens are treated as positive, consistent with the SUD literature [70]. We quantify treatment effects in terms of the difference in average cumulative days of substance use at 3-, 6-, 9-, and 12-month follow-up visits. The secondary substance use outcomes include the cumulative: (a) proportion of positive urine drug screens at 3-, 6-, 9-, and 12-months [71]; and (b) days of self-reported substance use at 3-, 6-, 9-, and 12 months. These analyses also leverage LTMLE and Super Learning.

The analyses of the primary PrEP and substance use endpoints involve two-sided hypothesis tests at the 0.05 level. We control for multiplicity of testing across the secondary endpoints [72]. For all primary and key secondary analyses, we report the point estimate in each intervention group, as well as estimated intervention effects, accompanied by appropriate confidence intervals. The clinical significance (as judged by the magnitude of the point estimate and width of the accompanying confidence interval) of our findings are described in addition to the statistical significance.

*Specific Aim 2: Estimate the cost of implementing and sustaining each intervention (Stepped Care with: a] A.S.K.-PrEP + CM; b] CM alone) and conduct a cost-effectiveness analysis to determine the value of each intervention relative to SOC, and to each other, from the healthcare-sector, state-policymaker, and societal perspectives.*

The economic analysis is conducted using well-established guidelines, from the healthcare-sector, state-policymaker, and societal perspectives [73, 74]. First, the resources and associated costs required to implement and sustain each intervention (*A.S.K.-PrEP* stepped to *A.S.K.-PrEP* + CM; *A.S.K.-PrEP* stepped to CM alone) are estimated using a detailed microcosting analysis. Second, we estimate the value of each intervention relative to SOC, and each other, including extrapolating the downstream savings resulting from improvements in SUD, and concomitant reductions in related risk behaviors. The resource costing method is used to value resources, including those identified for implementation/sustainment of the intervention, and those utilized by participants, by perspective [75]. Unit costs are derived from sources reflecting national “real-world” costs faced by the relevant stakeholders, and are adjusted for inflation [74].

The outcome of the cost-effectiveness analysis is the incremental cost-effectiveness ratio (ICER), which is calculated as the incremental cost of a given intervention relative to an alternative, divided by the incremental mean effectiveness of the two interventions. The primary measure of effectiveness for the economic evaluation is QALYs; the second is advancement along the PrEP Care Continuum. The QALY is a measure that combines the HRQoL associated with an individual’s health state and their time spent in that state, and is the foremost effectiveness measure in economic evaluation studies due to its comparability across interventions/disorders, and the existence of generally-accepted value thresholds [74, 76]. The PrEP Care Continuum is an important and widely accepted model/tool for assessing PrEP care outcomes at both an individual and a public-health level; thus, the additional cost required to achieve a one-step increase along the Continuum for the average trans woman or SMM with an SUD, has important clinical and policy implications. Two ICERs (one for each effectiveness measure) are calculated for each stakeholder perspective at both 6 months (intervention completion; immediate effects) and over the 12-month observation period (intervention + follow-up).

ICERs consist of predicted mean cost and effectiveness values. To help address censored data we model the person period and estimate all regressions using a multivariable generalized linear mixed model (GLMM) [74]. Separate multivariable GLMMs are estimated to predict the mean dollar value for each resource, the health utility value, and PrEP Care Continuum steps gained at each time period, by study arm. Predicted mean costs and steps gained are then be summed and tested over the relevant time periods (6 months, 12 months), and QALYs gained are estimated using the predicted health utility values and the area under the curve methodology, then tested [74]. Standard errors are estimated via nonparametric bootstrapping techniques within the multivariable framework. Methods to reduce bias from participant attrition and missingness are combined within the non-parametric bootstrap, following recommended approaches [67].

For each effectiveness measure, we determine which intervention is most cost-effective using the rules of strong and extended dominance. Finally, we estimate acceptability curves for each ICER, which illustrate the probability that an intervention is cost-effective for different value thresholds [77].

*Secondary Aim. Determine individual effects of specific substances, routes of administration, severity of SUD, social and structural determinants of health, and differing individual-level characteristics as moderators of outcomes.* As with Specific Aim 1, we utilize LTMLE to estimate subgroup-specific treatment effects based on participant-level characteristics. The magnitude of intervention effects are compared across relevant subgroups to infer moderation of intervention outcomes. We use Benjamini-Hochberg corrections to account for multiple testing and control for the false discovery rate of moderators.

*Exploratory Aim: Evaluate intervention engagement (i.e., number of A.S.K.-PrEP sessions in the initial 3-months) and intervention response (responders vs. non-responders) by chosen PrEP modality (i.e., oral daily or long-acting injectable).* We compare unadjusted response rates between, and average levels of engagement across the PrEP modalities using descriptive statistics. We also present covariate-adjusted analyses that account for participant-level differences. Because this analysis involves self-selected medication adherence, we control for potential confounders of PrEP modality and response. We use the theory of directed acyclic graphs to determine an appropriate set of confounders, and TMLE to estimate the impact of the PrEP modalities on response. We use controlled direct effects to quantify the impact of intervention engagement as a mediator of intervention response and determine whether there is a differential impact of PrEP modality on response after controlling for engagement [78]. Causal sensitivity analyses are used to examine the robustness of our findings to the assumption of no unmeasured confounding.

## Results

Recruitment and enrollment began in May 2023. Recruitment spans approximately 36 months (approximately 7 enrolled participants/month, final *N* = 250). Enrollment goals are approximately 2–3 trans women/month and 4–5 SMM/month. Data collection, including all follow-up assessments, is expected to be completed in April 2027.

## Discussion

The *A.S.K.-PrEP* study is designed to determine the optimal (in terms of efficacy and cost-effectiveness) intervention of PrEP initiation, adherence, and persistence among trans women and SMM with an SUD. Because SUD is a major barrier to PrEP access, adherence, and persistence, increasing attention on the SUD using CM via a Stepped Care approach if the targeted milestone is missed, or maintaining the intervention if the milestone is met, should maximize the primary outcomes of PrEP initiation, adherence, and persistence, while minimizing participant burden and costs.

### Limitations

There are several challenges to the *A.S.K.-PrEP* clinical trial. First, substance use, housing instability, engagement in sex work, and other individual-level, social, and structural disparities may interfere with study participation. To address this, the first level of the *A.S.K.-PrEP* intervention in the Stepped Care approach was designed to reduce/remove barriers and link participants into behavioral health services. Second, episodes of short-term incarceration can impact study participation and follow-up assessment rates. Due to actual or perceived participation in the street economy and/or minor infractions, study participants may experience cycles of brief incarceration. Study staff monitor the LAC public records database for participants who miss appointments. When an incarcerated participant is found, we begin a mail correspondence with the individual. Third, some participants may lack identification necessary to obtain PrEP. The PrEP navigators help participants fill out documentation and accompany them to the Department of Motor Vehicles to get a free or low-cost ID with a wavier form. Fourth, most participants do not have health insurance, including Affordable Care Act coverage. Again, the PrEP navigators and the partnering PrEP care clinics assist participants with enrollment in public or private insurance or patient assistance programs. Some participants may lose or sell their oral-daily PrEP due to lifestyle needs (“diversion”). In recognition of this, PrEP adherence is stressed through a participant-centered treatment plan, including an analysis of how PrEP adherence outweighs selling PrEP. Furthermore, this study is conducted in an urban setting on the West Coast and may not be representative of the HIV and substance use co-epidemic, and the impacted communities in other regions of the United States. Additionally, due to limited resources, substance use frequency is only assessed with a 14-day recall period at each follow-up assessment visit, and weekly with a 7-day recall period for those re-randomized into the Contingency Management intervention. Finally, the sample may also be subject to self-selection bias, namely trans women and SMM who are more receptive to participating in a clinical trial or receiving services for HIV prevention and substance use reduction.

## Conclusion

Trans women and SMM with an SUD have the two highest HIV prevalence rates in the United States, which underscores the urgent need for effective measures to develop scalable behavioral interventions that can encourage PrEP initiation and advancement along the PrEP Care Continuum. Given the severe personal, population-health, and economic consequences associated with HIV acquisition, and the extent to which they are compounded by SUD [40, 41], there is a critical need for efficacious and cost-effective interventions to promote PrEP initiation, adherence, and persistence in trans women and SMM. The *A.S.K.-PrEP* study is unique, and contributes to the PrEP landscape literature, in that all eligible participants must have a verified DSM-5 SUD, and that a cost-effectiveness analysis is an integral component and a Specific Aim of the research. This rigorous clinical trial will provide data on scalable and effective PrEP strategies that could have a major public health impact and usher in a new model for PrEP delivery among trans women and SMM populations.

## Data Availability

Not applicable.

## Abbreviations

ARTAS: Anti-Retroviral Treatment and Access to Services
A.S.K-PrEP: Assistance Services Knowledge-PrEP
CAB: Community Advisory Board
CDC: Centers for Disease Control and Prevention
CM: Contingency Management
DATCAP: Drug Abuse Treatment Cost Analysis Program
DBS: Dried Blood Spot
DSM-5: Diagnostic and Statistical Manual-5
EHR: Electronic Health Record
GLMM: Generalized Linear Mixed Model
HIV-ASES: HIV Treatment Adherence Self-Efficacy Scale
HRQoL: Health-related Quality-of-Life
ICER: Incremental Cost-effectiveness Ratio
LAC: Los Angeles County
LTMLE: Longitudinal Targeted Minimum Loss-based Estimation
NBA: Needs and Barriers Assessment
NBA-Lite: Needs and Barriers Assessment-Lite
NMOS: Non-study Medical and Other Services
PrEP: Pre-Exposure Prophylaxis
PROMIS: Patient-Reported Outcomes Measurement Information System
PROPr: Patient-Reported Outcomes Measurement Information System-Preference
PWH: Persons with HIV
QALY: Quality-adjusted Life-year
SMM: Sexual Minority Men
SMS: Short Message Service
SOC: Standard of Care
SUD: Substance Use Disorder
TAF/FTC: Tenofovir Alafenamide/Emtricitabine
TDF/FTC: Tenofovir Disoproxil Fumarate/Emtricitabine
TLFB: Timeline Follow-Back
